# Noninvasive Classification of Glioma Subtypes Using Multiparametric MRI to Improve Deep Learning

**DOI:** 10.3390/diagnostics12123063

**Published:** 2022-12-06

**Authors:** Diaohan Xiong, Xinying Ren, Weiting Huang, Rui Wang, Laiyang Ma, Tiejun Gan, Kai Ai, Tao Wen, Yujing Li, Pengfei Wang, Peng Zhang, Jing Zhang

**Affiliations:** 1 Department of Magnetic Resonance, Lanzhou University Second Hospital, Lanzhou 730030, China; 2 Second Clinical School, Lanzhou University, Lanzhou 730000, China; 3 School of Mathematics and Statistics, Lanzhou University, Lanzhou 730000, China; 4 Department of Clinical Science, Philips Healthcare, Xi’an 710000, China; 5 Department of Pathology, Lanzhou University Second Hospital, Lanzhou 730030, China

**Keywords:** ADC, gliomas, WHO CNS5, deep learning

## Abstract

**Background**: Deep learning (DL) methods can noninvasively predict glioma subtypes; however, there is no set paradigm for the selection of network structures and input data, including the image combination method, image processing strategy, type of numeric data, and others. **Purpose**: To compare different combinations of DL frameworks (ResNet, ConvNext, and vision transformer (VIT)), image preprocessing strategies, magnetic resonance imaging (MRI) sequences, and numerical data for increasing the accuracy of DL models for differentiating glioma subtypes prior to surgery. **Methods**: Our dataset consisted of 211 patients with newly diagnosed gliomas who underwent preoperative MRI with standard and diffusion-weighted imaging methods. Different data combinations were used as input for the three different DL classifiers. **Results**: The accuracy of the image preprocessing strategies, including skull stripping, segment addition, and individual treatment of slices, was 5%, 10%, and 12.5% higher, respectively, than that of the other strategies. The accuracy increased by 7.5% and 10% following the addition of ADC and numeric data, respectively. ResNet34 exhibited the best performance, which was 5% and 17.5% higher than that of ConvNext tiny and VIT-base, respectively. **Data Conclusions**: The findings demonstrated that the addition of quantitatively numeric data, ADC images, and effective image preprocessing strategies improved model accuracy for datasets of similar size. The performance of ResNet was superior for small or medium datasets.

## 1. Introduction

Molecular biomarkers of gliomas provide important information regarding the diagnosis and prognosis of gliomas [1,2]. According to the new WHO Classification of Tumors of the Central Nervous System (WHO CNS5, Geneva, Switzerland, 2021), diffuse gliomas are categorized into three subgroups based on mutations in isocitrate dehydrogenase (IDH), 1p19q-codeletion status, and mutations in telomerase reverse transcriptase (TERT) (astrocytic, IDH-mutant; oligodendroglial, IDH-mutant, and 1p/19q-codeleted; glioblastoma (GBM), IDH-wildtype) [3]. Previous studies have demonstrated that compared to IDH-wildtype gliomas, IDH-mutant gliomas are less aggressive and have a better response to treatment with temozolomide [4]. Similar to the IDH mutants, the 1p/19q-codeleted subtype responds well to certain combinations of chemotherapy and is associated with a better prognosis [5]. Therefore, understanding the molecular type of gliomas is essential for providing prognosis information and selecting the most appropriate treatment regimen.

Genetic information regarding gliomas is currently obtained by analysis of biopsied or surgically resected tumor tissues by neuropathologists. However, analysis of the same sample by different experts is likely to result in inconsistencies even when strict grading strategy standards are followed, owing to the heterogeneity of the tumor itself and the extraction of atypical samples [1]. Additionally, molecular genetic testing approaches are usually expensive and time-consuming, and all medical institutions are not capable of providing professional testing services. Noninvasive imaging techniques that provide supplementary information for treatment decisions can aid doctors in explaining the prognosis for some patients who cannot undergo surgery.

Magnetic resonance imaging (MRI) allows the noninvasive evaluation of patients by including the genetic information of the local region and by revealing the heterogeneity of the global area. Numerous studies have attempted to use MRI imaging in recent years for solving virtual biopsies, and deep learning (DL) proves to be a successful approach in this regard.

DL is a subset of machine learning, and is essentially a neural network with multiple layers. It has four main aspects in medical image analysis tasks, including classification, segmentation, detection, and registration [6,7]. The network structure of the classification task primarily includes convolutional neural networks (CNNs) and non-convolutional vision transformers (VITs). Studies on the molecular typing of gliomas have employed residual convolutional neural networks (ResNet) to achieve superior results, and the accuracy of the overall test set is reported to be greater than 85% [8,9,10]. The unique residual block of ResNet deepens the network without lowering the effect [8]. These studies additionally demonstrated that the establishment of a three-classification model aids in increasing model accuracy by lowering the accumulation of errors in numerous two-step strategies. The studies further confirmed that the shortcomings of traditional MRI may be compensated by ADC imaging, which can provide insights into tumor infiltration in a narrow range [9]. However, the lower-level features outside the effective receptive fields in CNNs cannot be represented owing to the fact that higher-level feature maps only describe the characteristics within those fields. The attention mechanism of VITs has therefore been employed for solving this problem. VITs divide an image into a series of non-overlapping small blocks, which are analyzed as elements, similar to words [11]. It has been demonstrated that the performance of VITs is superior in certain X-ray, computed tomography (CT), and MRI classification tasks [12,13,14]. It has been recently demonstrated that the performance of the ConvNext network, a CNN network inspired by the swin-transformer parameter setting, is comparable to the VIT model, with the ImageNet-22K dataset [15]. The network performed well in predicting breast tumors with ultrasound (US) imaging data and the detection of COVID-19 using lung CT imaging data [16].

Although comprehensive predictive models have been described in previous studies, there are several issues with DL applications that need to be resolved. At present, there are no definite data-slicing methods from the input side, and the accuracy of the models is affected by the permutation and combination of different MRI sequences. Additionally, the incorporation of numeric data, including clinical information and known hallmark features of each subtype, such as patient age and gender, tumor position, and T2 Fluid Attenuated Inversion Recovery (T2-FLAIR) mismatches, can improve the predictive ability of the models. The architecture of the neural network also plays an important role in determining the classification effect.

In this study, we compared different DL frameworks, MRI sequences, and slice preprocessing strategies for improving the accuracy of our model, and aimed to explore the effect of different inputs on the accuracy of our model in predicting glioma subtypes. Numeric mixed data extracted from clinical information and known hallmark features were also added as extra feature inputs for predicting glioma subtypes.

## 2. Materials and Methods

### 2.1. Patients

**Figure 1:**
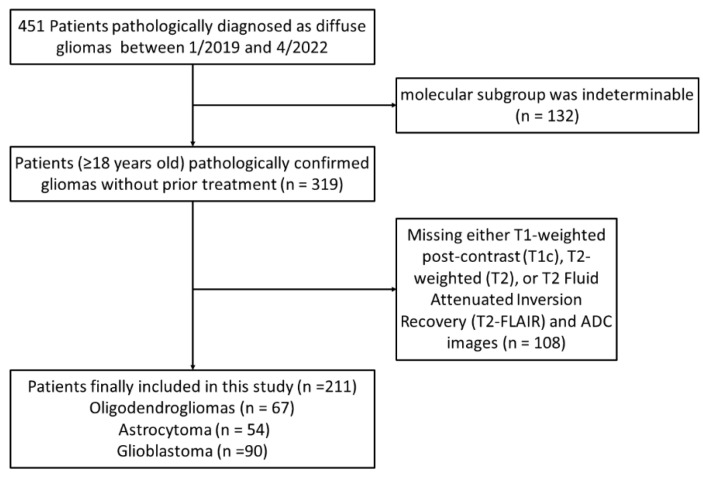
Flowchart depicting patient inclusion and exclusion criteria.

This retrospective study was approved by the local institutional review board and informed consent was waived. A total of 451 patients with brain tumors were recruited from Lanzhou University Second Hospital China, between January 2019 and April 2022. Patients with the following inclusion criteria were selected for the study: (1) patients ≥ 18 years old; (2) patients with pathologically confirmed gliomas; and (3) patients who did not undergo prior treatment. The exclusion criteria were as follows: (1) patients with indeterminable molecular subgroups (n = 132); (2) patients for whom preoperative MRI acquisitions did not include either T1-weighted post-contrast (T1c), T2-weighted (T2), or T2-FLAIR and ADC imaging (n = 108). A flowchart of the strategy used for patient inclusion/exclusion in this study is provided in Figure 1. A total of 211 patients were finally included in the study.

### 2.2. Pathological Analysis

The operative tissue samples were processed using standard clinical techniques. The IDH1 mutation status was assessed by immunohistochemistry analysis using the H09 clone (Dianova, Hamburg, Germany) generated against the R132H mutant of IDH1. Fluorescent in situ hybridization testing was performed for assessing 1p/19q codeletion. The TERT promoter mutation status was assigned by sequencing. All the cases were evaluated and re-classified according to the WHO CNS5 criteria by two experienced pathologists who were blinded to the findings of imaging. The final decision was made by a third pathologist in case of any discordance in the results.

### 2.3. MRI Protocols

MRI imaging was performed using a 3T scanner (Ingenia and Ingenia CX; Philips Healthcare, Best, The Netherlands) equipped with 80/200 mT/m gradients and a dS Head 16 channel-coil. The imaging parameters of the MRI sequence protocols are summarized in Table S1. The imaging protocol included T2WI and T2-weighted FLAIR, along with 3D T1-weighted IR-SPGR imaging, which performed the pre- and post-injection of a gadolinium-based contrast agent. A 2D trace-weighted single-shot echo planar imaging sequence (TR/TE: 2668/88 msec, slice thickness 6 mm, matrix 144 × 192, number of acquisitions: 5, acquisition time: 32 s, flip angle: 90°) was used for diffusion-weighted imaging. The ADC map was automatically computed with 2 gradient values (b = 0 and b = 1000 s/mm^2^) by the processing software provided with the MRI scanner.

### 2.4. Dataset

The three glioma subtypes were randomly divided at a ratio of 7:1.5:1.5 into the training, validation, and test sets, respectively. It was ascertained whether the proportions of the three tumors in the training, validation, and test sets matched those of all the patients. The final training, test, and validation sets comprised 139, 32, and 40 patients, respectively. (Table 1).

### 2.5. Image Postprocessing

**Figure 2:**
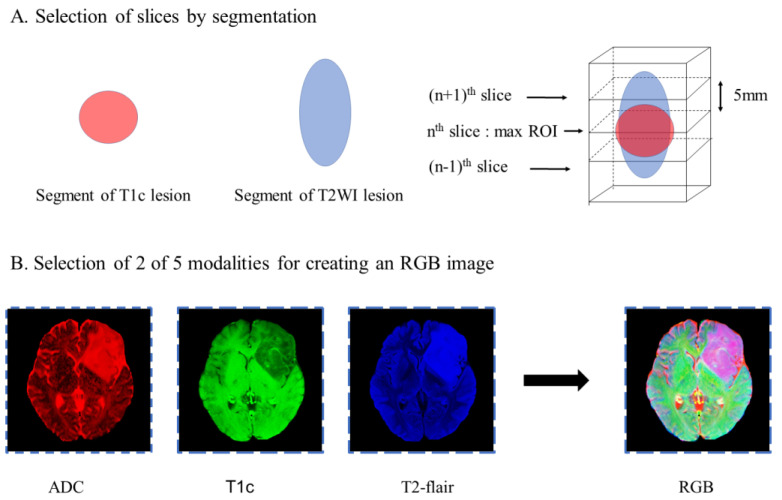
Schematic depicting the image processing strategy. (**A**) The largest tumor area and two adjacent layers 5 mm apart in the axial direction were selected using segmented contrast-enhancing (CEL) or T2 lesions (T2L). (**B**) Three of the four sequences of interest (T2-FLAIR, T1c, T2, and ADC) were placed in the red (R), green (G), and blue (B) channels of a color image that was used as the input to the network.

All the images were registered to the T1 image volumes using the BRAINSFit or Elastix tool of 3D Slicer with B-spline warping, and resampled to an identical 1 mm isotropic spatial coordinate (Figure 2).

The signal intensities were subsequently normalized across all the images, as described hereafter. Firstly, the images in the skull stripping method were obtained by manually delineating the ROI. Secondly, pixels above the 99.9th percentile were selected as threshold pixel intensity and denoted as the 99.9th percentile. Thirdly, the mean of each pixel was removed, and the resulting value was divided by the standard deviation. Fourthly, the images have scaled a value between 0 and 1 by removing the minimum and dividing by the gap between the minimum and maximum pixels.

The image slice with the largest tumor area in the axial direction was selected by masks of the 3D segmented lesion volumes of T2WI and T1c. Additional slices spaced 5 mm apart were added once to the two adjacent layers of the biggest tumor area in the axial direction. The images were not cropped for better accuracy.

Different combinations of the 3 image modalities obtained from each of the three groups described in Figure 2 were subsequently used to produce a multi-contrast RGB image for each slice, where each image contrast was preserved as a red, green, or blue color channel, similar to the method described by Julia et al. [17] The multi-contrast RGB image thus constructed enabled transfer learning from a pre-trained network with exceptionally high accuracy in classifying a large-scale dataset of millions of color images in ImageNet.

### 2.6. Numeric Data

The numeric data included the age and gender of the patients, tumor position, T2-FLAIR mismatches, gadolinium enhancement, and tumor margins. The age of the patient at the time of surgery was also included as numeric data.

The position of a tumor was based on the coordinates of its center, and was divided into three groups: the first group included tumors in the frontal, parietal, or occipital lobes; the second group included tumors in the temporal and insular lobes; and the third group included tumors in other regions. Gadolinium enhancement was classified as non-enhancement, patchy enhancing, or rim enhancing.

T2-FLAIR mismatch signs were indicated by the presence or absence of complete/near-complete hyperintense signals on T2WI and relatively hypointense signals on FLAIR except for a hyperintense peripheral rim.

The tumor margin was selected as an indicator of a strong predominance of tumor character; sharp or blurred tumor interfaces with the brain were observed on both T1- and T2-weighted sequences. Tumor margin circumscription was judged as a summary marker from all pulse sequences, and was considered sharp if >50 percent of the tumor circumference was geographically marginated, as if a pencil line could be traced around the tumor. Circumscription was deemed absent if 50 percent of the tumor circumference was circumscribed [9,18].

The aforementioned numeric data were analyzed by two neuroradiologist readers who were blinded to the histopathologic diagnosis, molecular classification, and patient outcome. Inter-reader agreement was determined following the collection of independent data, and discordant results were resolved through consensus.

The categorical variables, including the gender of the patients, tumor position, gadolinium enhancement, T2-FLAIR mismatch signs, and tumor margins, were one-hot encoded.

### 2.7. Model Details

#### 2.7.1. Slice Preprocessing Strategies

**Figure 3:**
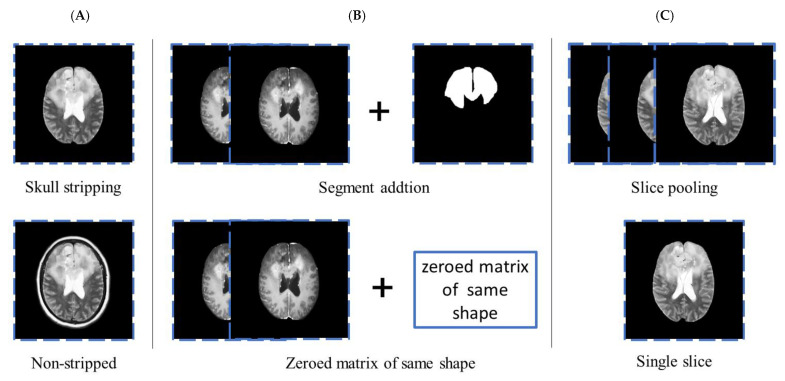
Comparison of different image preprocessing strategies. (**A**) skull stripping or non-stripped strategies. (**B**) Segment addition or replacement by a zeroed matrix of the same shape. (**C**) Slice pooling for a single prediction per patient or individual treatment of each slice.

Three groups were set up for comparing the image processing strategies and determining the optimum approach that can be applied for improving model accuracy. Group A and group B independently investigated whether skull stripping and lesion segments, which contain location information but lack image information, would improve prediction accuracy. Group C investigated two different slice-combining paradigms, of which the first paradigm involved the individual treatment of each slice during training and combining slice predictions later on, as described by Chang et al. [19]. The second strategy involved the pooling of slices for a single prediction per patient, as described by Bien et al. (Figure 3) [20].

#### 2.7.2. Structures

**Figure 4:**
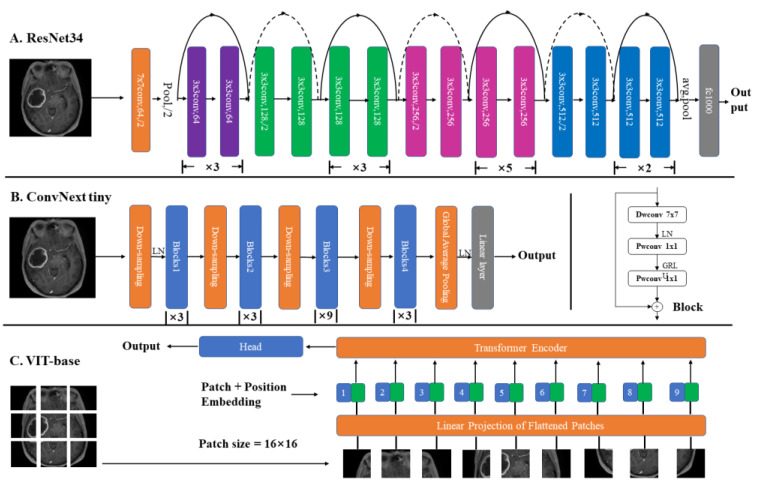
Structure of different networks. (**A**) the structure of ResNet34. The mark under the block indicates the number of repetitions of the block in this group. (**B**) the structure of ConvNext. The block structure could be seen on the right, which consists of two pointwise (PW) convolutions and a depthwise convolution (DW) convolution, seen on the right. (**C**) the structure of Vit. Contains Patch + Position Embedding and Transformer Encoder.

As depicted in Figure 4, 3-class models of DL were developed for subtyping diffuse gliomas based on ResNet34, ConvNext tiny, and VIT-base. 

**Figure 5:**
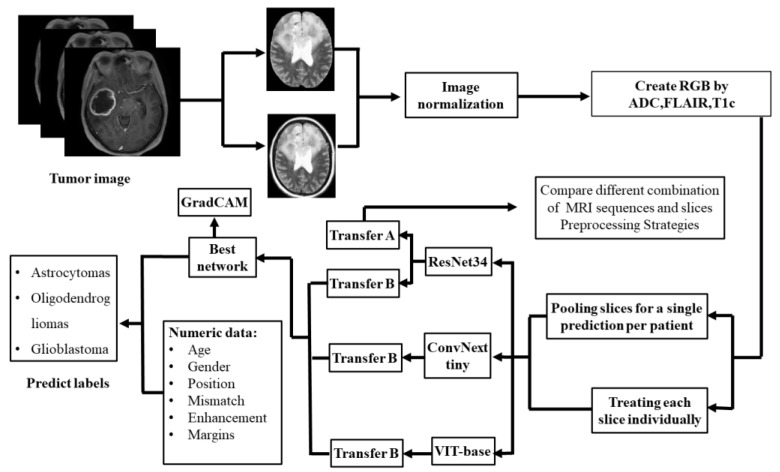
The flowchart of the entire process. All images were first normalized. Secondly, ADC, FLAIR, and T1c were used to create RGB. Thirdly, we used the second strategy of transfer learning to compare three frameworks and select the best one. The best model was used in combination with numerical data to get the final prediction. ResNet34 with the first strategy of transfer learning was used to compare the different MRI sequence and slicer preprocessing strategies.

ResNet34, ConvNext tiny, and VIT-base were pre-trained on ILSVRC2012 datasets (ImageNet-1K), and two strategies of transfer learning were used for training, as described hereafter [21]. In the first strategy, a fully connected layer was added to the original network, while the second strategy involved the retraining of fully connected layers. Only image data were used for training the models. The first strategy of transfer learning is used in that use only image inputs, while the second strategy is used on models using both image and numeric data inputs. The different combinations of MRI sequences and slice preprocessing strategies were compared using ResNet34 and the first transfer learning strategy. The performance of the different networks was compared using the same combination of MRI sequences and the second transfer learning strategy. The networks with the best performance integrated the numeric data to generate the final classification result. The flowchart of the entire process is depicted in Figure 5.

#### 2.7.3. Model Explanation

GradCAM, a heatmap-based feature attribution method, was used to explain the model [22]. In contrast to CAM, GradCAM does not require modification of the network structure and has been validated in the literature on DL for assigning feature importance to different areas of images [17,23]. By extracting features in areas corresponding to human interpretation, this method rapidly confirmed whether the models constructed herein were behaving as expected.

#### 2.7.4. Model Evaluation

The performance of the models was assessed by evaluating the accuracy using the test sets. The accuracy was determined using the following formula:

$$Total\;accuracy=\frac{Total\;correct\;predictions}{Total\;number\;of\;predictions}\times 100\%$$

$$Class\;accuracy=\frac{Class\;correct\;predictions}{Class\;number\;of\;predictions}\times 100\%$$

We further employed receiver operating characteristic curve (ROC) analysis to assess the diagnostic performance of different networks (Supplement Figure S1). 

A heatmap-based feature attribution method, GradCAM [22], was used for model explanation. L1 regularization was employed in the final feature layer for improving visualization. The train and loss curve of the combination of ResNet34 and the numeric data model was additionally determined (Supplement Figure S2).

## 3. Results

### 3.1. Patient Characteristics

The clinical characteristics of the patients are provided in Table 2. The patients were categorized into the astrocytoma (n = 54), oligodendroglioma (n = 67), and glioblastoma (n = 90) groups. The patients had a mean age of 48.1 ± 11.8 years, and the mean age of the patients in the astrocytoma group (40.3 ± 11.5 years) was lower than that of the other subtypes.

### 3.2. Model Comparison

#### 3.2.1. Addition of ADC to Models

We evaluated whether the addition of ADC would improve the utilization of the simple common sequences as inputs to the ResNet34 model. The model with the best performance had an overall patient accuracy of 60.0% with T1c and FLAIR images and test sets, and the individual class accuracies for the oligodendroglioma, astrocytoma, and glioblastoma subtypes were 78.6%, 50.0%, and 62.5%, respectively. However, the overall test accuracy of the model with the best performance increased by 7.5% when ADC maps were combined with T1c and FLAIR images in the input. The combination of T1, T2, and T1c resulted in the same overall test accuracy as that of the combination of T1, T2, and ADC. Details of the class accuracies for each test cohort and the confusion matrices are provided in Table 3.

#### 3.2.2. Different Slice Preprocessing Strategies

We investigated the effect of different slice preprocessing strategies on model performance. The overall patient accuracy of the skull stripping group was 67.5% with the test set, and the individual class accuracies for the oligodendroglioma, astrocytoma, and glioblastoma subtypes were 85.7%, 40.0%, and 62.5%, respectively. The overall patient accuracy of the skull-stripping group was 5% higher than that of the non-stripped group (Table 4). Furthermore, the overall test accuracy increased by 10.0% following a segment addition to the input. However, slice pooling resulted in an overall patient accuracy of 42.5% with the test set, which was 12.5% lower than that of the individual slice treatment approach (Table 4).

#### 3.2.3. Benefits of Using Numeric Data

**Figure 6:**
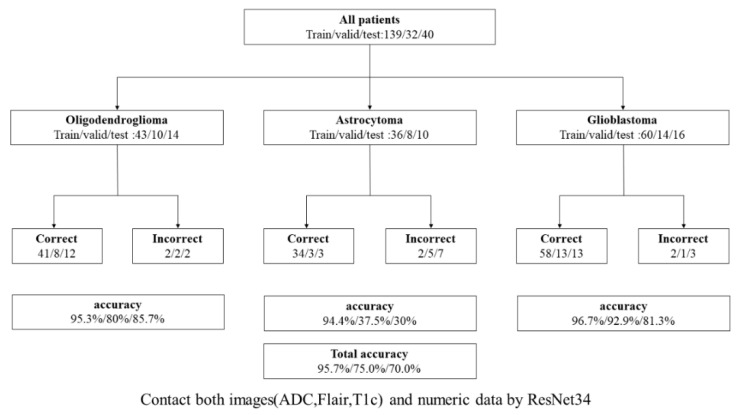
Patient accuracy and class accuracies of the final models. The 3-class model based on ResNet34, and integrated both image and numeric data, was selected as the best-performing model.

The use of both image and numeric data inputs in the best 3-class model based on ResNet34 resulted in an overall patient accuracy of 95.7%, 75.0%, and 70.0% for the training, validation, and test sets, respectively, while the individual test class accuracies were 85.7%, 30%, and 81.3% for the astrocytoma, oligodendroglioma, and glioblastoma subtypes, respectively. The test class accuracy of this strategy was 2.5% higher than that of the best 3-class model using image data only. The 3-class model based on ResNet34, and integrated both image and numeric data, was selected as the best-performing model (Figure 6).

#### 3.2.4. Comparison of Different Networks

When using ResNet34, the first strategy of transfer learning had an overall accuracy of 67.5%, and outperformed the second retraining strategy with an overall accuracy of 60.0%. Furthermore, the ResNet34 method outperformed ConvNext tiny and VIT-base in terms of overall accuracy and prediction of glioma subtypes (Table 5).

#### 3.2.5. Visualization and GradCAM

**Figure 7:**
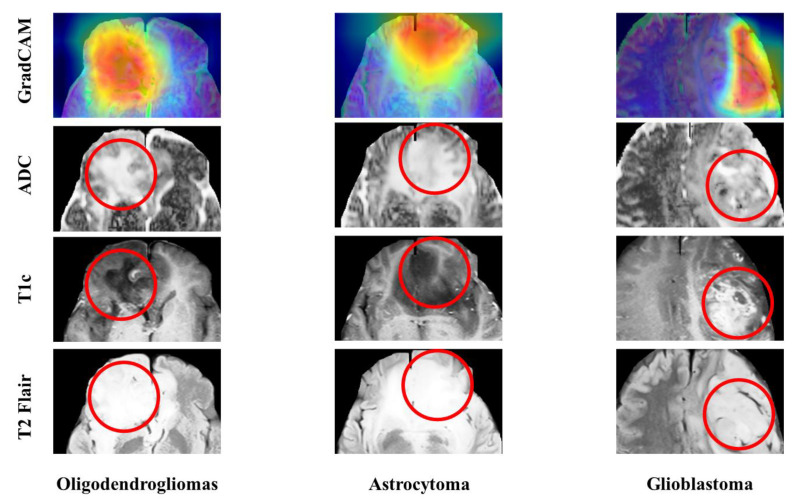
Visualization of imaging features and GradCAM analysis of the final 3-class model for predicting oligodendrogliomas, astrocytomas, and glioblastomas. The regions of red cycles represent the lesions. Colors nearer to red and blue indicate regions with higher and lower weights.

Figure 7 depicts the representative GradCAM images of the correct and incorrect predictions of the best 3-class model using ADC, T1c, and T2-FLAIR images as inputs. The regions of red cycles represent the lesions. In GradCAM imaging, colors nearer to red and blue indicate regions with higher and lower weights, respectively, in the network. The network focuses on lesions in the majority of correctly predicted tumors, while GradCAM heatmaps aid in identifying whether the network is not looking at the correct region of images from misclassified patients.

## 4. Discussion

In this study, we thoroughly explored and compared different combinations of DL frameworks, MRI sequences, slice preprocessing strategies, and numerical data for simultaneously differentiating astrocytomas, oligodendrogliomas, and glioblastomas prior to surgery. Model accuracy differs based on the slice preprocessing approach used. In this study, we primarily compared the effects of three different slice processing strategies, namely, skull stripping, segment addition, and slice-combining paradigms. Although skull stripping has been employed in the majority of previous studies, few studies have achieved superior results using this strategy. The results demonstrated that the overall patient accuracy of skull stripping was 5% higher than that of the non-stripped group. This could be attributed to the fact that the final algorithm comprised learning features derived from the skull and not from an area of the brain. Similar to the method described by Matsui et al. [24], we included a segment in addition to an imaging sequence; however, our segments only contained positional information. The addition of segments increased the accuracy by 10%, and increased attention to the position of the brain segments. Two different slice-combining paradigms were compared, of which the first paradigm included the individual treatment of each slice while training and combining slice predictions later on, and the second strategy involved pooling slices for a single prediction per patient. The first strategy treats each slice independently; therefore, a single batch frequently contains slices from multiple patients. The gradients are backpropagated slice by slice and a final patient-level prediction is obtained only after the completion of training. In contrast, the second method uses all slices from a single patient in the same batch; therefore, the number of slices represents the effective batch size. Average or maximum pooling is performed in the final layer for condensing all the slices into a single feature vector that generates a single prediction per patient. A single value is therefore back-propagated through the network for each patient after the calculation of losses. It has been reported that updating network weights based on individual slices improves training/validation loss curves and increases overall patient-level accuracy [17]. The 3-class model approach was selected owing to the prior superiority of its performance compared to that of the 2-tiered strategy, which first predicts glioblastomas, and subsequently predicts astrocytomas and oligodendrogliomas. In this instance, the accumulation of errors during a repeated 2-group classification is worse compared to the complications of 3-group classification [17,25].

One unanticipated finding of this study was that the ResNet34 architecture achieved higher accuracy than ConvNext tiny and VIT-base. The lower accuracy of ConvNext tiny could be attributed to the fact that the benefit of the depth of the neural network of the tiny architectures of ConvNext tiny and its depthwise convolution are often not observed until the higher-order data of greater magnitude are used for training. Previous studies have demonstrated that the classification accuracy of the VIT-base is equivalent to Resnet only when the size of the images is scaled to 384 × 384, and the convolutional neural network outperforms VIT-base when the size of the images is 224 × 224 [26]. The number of parameters and flops in ResNet34 is substantially lower than those of the VIT-base and Convnext tiny, which allow significant savings in computational resources. Additionally, Khan et al. [27] presented a model that extracted features using two separate CNN networks at the same time, and the model outperformed a single CNN network. However, as mixed CNN network structures require retraining and cannot use the pre-trained weights of ImageNet-1K, no comparison was performed in this study.

ADC performed best when combined with T1c and T2-FLAIR, and the overall test accuracy increased when the generic sequences were included. ADC predicted the complicated diffusion patterns within a voxel of biological tissue. Cellular proliferation increases membrane density and raises the membrane surface; the volume of slow diffusion phases (SDP) in each voxel consequently increases almost linearly with the number of cells per voxel, resulting in a lower ADC following cellular proliferation [28,29]. Recent studies have suggested that ADCs can aid in determining the IDH mutation status; however, ADCs did not provide the advantage of subtype prediction over T1c alone in the present study. The findings indicated that T1c is indispensable in predicting glioma subtypes.

The results of the present study implied that the majority of DL models could precisely discriminate glioblastomas from oligodendrogliomas in the multiclass setting. The earlier result could be explained by the fact that the IDH family of enzymes, which exist in glioblastomas, are part of the Krebs cycle and therefore present in the cytoplasm. Intermediates of the Krebs cycle are used in anabolic reactions that lead to the biosynthesis of various substances, including nucleotides, phospholipids, amino acids, and choline, provide building blocks for cellular proliferation, and act as precursors for membrane biosynthesis, all of which support tumor growth and metastasis [30,31,32,33,34,35]. As IDH-wildtype tumors have more vasculogenesis than IDH-mutant tumors, it follows that IDH-wildtype tumors have higher microvascular density [36,37]. These alterations can be easily observed in anatomical and functional sequences; however, the accuracy of glioblastoma prediction in this study was slightly lower than that reported in previous studies [17,38]. According to CNS5, glioblastomas may be designated as IDH-wildtype CNS WHO grade 4 even in cases that are apparently of histologically lower grade and where images may lack the obvious features of high-grade glioblastoma [3]. Furthermore, oligodendroglial tumors are highly cellular lesions with densely packed, relatively small cells in the central region. Diffuse infiltrative growth is frequently associated with the formation of prominent secondary structures in the peripheral regions with low cellular density, including the clustering of tumor cells around pre-existing neuron perikarya (satellitosis), under the pial surface (subpial aggregation), and surrounding small cortical vessels (perivascular aggregates) [39]. Both ADC and T1 can accurately reflect the aforementioned characteristics of oligodendrogliomas; however, the power of DL models in discriminating astrocytomas is still limited even after the addition of numeric data. This is attributed to the fact that the majority of astrocytomas have no fixed features. Although low-grade astrocytomas have a specific T2-FLAIR mismatch, the sensitivity of predictive models is low. Low grades are, therefore, frequently mislabeled as oligodendrogliomas, while high grades are mislabeled as glioblastomas. Certain gliomas have characteristics of both astrocytes and oligodendrocytes. The cells of the two subtypes may remain diffusely mixed or separated, although the latter form is rare. The biological diversity of these tumors is difficult to capture by precise microscopic criteria, and histopathological samples are not always completely representative [39,40]. Additionally, the field strength parameters of heterogeneous scanning can produce images of varying quality, and may play a crucial role in reducing the sensitivity.

The highest overall accuracy of 67.5% for subtype prediction was achieved when MRI data were included in the datasets. In contrast, an overall accuracy of 70.0% was achieved when both imaging and numeric data were incorporated. However, the addition of numeric data increased the accuracy by only 2.5%. Owing to the addition of clinical information, the second strategy of transfer learning should be used for retraining the fully connected layer; however, the best model using image data used the first strategy of transfer learning. The accuracy improved by 10% when the second strategy of transfer learning was used and clinical information was included. In fact, the addition of numerical data bridged the deficit in transfer learning. Owing to the limitation of transfer learning channels, only three different image sequences contributed to the network for training, which was insufficient for accomplishing the goal of multimodal assessment. However, model accuracy would remain very poor despite the use of more imaging and numeric data if transfer learning is not used. The numeric data attempts to secure the entry of all sequence information and implements multi-modal diagnosis when the transfer learning approach is used in networks. In this study, the numeric data included clinical information and the known hallmark features of each subgroup. Previous studies have demonstrated that glioma subtypes occur in fairly well-defined age groups [41]. The preferred tumor location also differs significantly across different glioma subtypes [42,43], and tumor margins in 1p/19q-codeleted tumors are frequently indistinct [9]. Another significant finding is the T2-FLAIR mismatch sign, which is 100% specific for the diagnosis of IDH-mutant 1p/19q non-codeleted gliomas (astrocytomas) [44]. These numerical characteristics distinguish the glioma subtypes from one another. Although the efficacy of feature extraction in DL largely depends on the scale of the dataset, additional numeric data with proven substantial differences across subtypes may further increase the accuracy when the sizes of the datasets are same.

It is generally accepted that a black box is not very interpretable for widely used DL networks, such as CNN. GradCAM heatmaps depict regions of the network that are prioritized for a given classification. The results of GradCAM analysis in this study demonstrated that DL models can learn from signals in tumor regions, and that it is possible to learn generalizable imaging features. The GradCAM heatmaps depicted in Figure 4 provide evidence that the network focused on the lesions in the majority of correctly predicted tumors. Although GradCAM heatmaps provide insights into the region that the model is looking at, they do not attribute feature importance and have limited spatial resolution based on the size of the final output layer of the selected model [17].

The present study has some limitations as described hereafter. First, the number of patients in this study was quite low for DL and more patients should be included for adequate training in future studies. Secondly, according to the new WHO CNS5 scheme, gliomas with IDH-wildtype and grade 2–3 histopathological types should have one of the following characteristics in order to be diagnosed as glioblastomas: high levels of epidermal growth factor receptor (EGFR), whole chromosome 7 gain and whole chromosome 10 loss (+7/−10), or mutations in the TERT promoter. However, only TERT promoter mutations were considered in the present study.

## 5. Conclusions

In conclusion, we explored the effect of different inputs on the accuracy of models predicting glioma subtypes. The results demonstrated that certain slice preprocessing strategies, including skull stripping, segment addition, and the individual treatment of slices could achieve superior results. Additionally, the inclusion of ADC improved the overall accuracy of our models, which emphasized the need for adding functional sequences that closely reflect the underlying tumor biology and can be used in future multisite investigations of glioma subtypes. The inclusion of extra quantitative numeric data with a validated substantial difference between subtypes also increased the accuracy when the datasets were of the same size. We believe that the inclusion of more clinical symptoms or radiomics features would provide more information to our model and improve classification outcomes. More functional sequences and numerical data need to be included in follow-up studies with larger datasets for validating our findings and providing insights into training CNN models for the classification of gliomas.

## Figures and Tables

**Table 1:** Summary of dataset.

	All Dataset	Training	Validation	Testing
All gliomas	211	139	32	40
Oligodendrogliomas	67	43	10	14
Astrocytoma	54	36	8	10
Glioblastoma	90	60	14	16

**Table 2:** Numeric data of the patients included in the study.

Parameter	All Gliomas	Oligodendroglioma	Astrocytoma	Glioblastoma
**Number of patients**	211	67	54	90
**Median age (years)**	48.1 ± 11.8	46.9 ± 10.0	40.3 ± 11.5	53.6 ± 10.4
**Gender**				
Male	105	32	24	50
Female	106	35	30	51
**Enhancement category**				
Nonenhancing	76	37	37	2
Patchy enhancing	47	28	13	6
Rim enhancing	88	3	3	82
**Tumor location category**				
Frontal, parietal, or occipital	139	57	36	46
Temporal and insular	50	7	10	33
Others	22	3	8	11
**T2-FLAIR mismatch**				
Present	14	1	13	0
Absent	197	66	41	90
**Tumor margin**				
Present	27	4	21	2
Absent	184	63	33	88

**Table 3:** Summary of classification results for different sequence combinations on test sets.

Combinations of 3 Image Modalities	n (Total)	n (Correct)	Accuracy	Subtype	Confusion Matrices		
***T1, T2, T1c***	40	20	50.0%		Oligodendroglioma	Astrocytoma	Glioblastoma
Oligodendroglioma	14	7	50.0%	Oligodendroglioma	7	0	7
Astrocytoma	10	3	30.0%	Astrocytoma	0	3	7
Glioblastoma	16	10	62.5%	Glioblastoma	3	3	10
***T1, T2, ADC***	40	20	50.0%				
Oligodendroglioma	14	4	28.6%	Oligodendroglioma	4	0	10
Astrocytoma	10	0	0%	Astrocytoma	4	0	6
Glioblastoma	16	16	100.0%	Glioblastoma	0	0	16
FLAIR, T1c, Zero*	40	24	60.0%				
Oligodendroglioma	14	11	78.6%	Oligodendroglioma	11	2	1
Astrocytoma	10	5	50.0%	Astrocytoma	5	3	2
Glioblastoma	16	10	62.5%	Glioblastoma	5	1	10
***FLAIR, T1c, ADC***	40	27	67.5%				
Oligodendroglioma	14	12	85.7%	Oligodendroglioma	12	0	2
Astrocytoma	10	4	40.0%	Astrocytoma	5	4	1
Glioblastoma	16	11	68.8%	Glioblastoma	5	0	11

Note: ResNet34 and second strategy of transfer learning were used to generate all result. Bold parts represent titles of different combinations. Zero *: zeroed matrix of same shape.

**Table 4:** Summary of classification results for different slice preprocessing strategies on test sets.

Image Processing Strategy	n (Total)	n (Correct)	Accuracy	Subtype	Confusion Matrices		
***Skull stripping (FLAIR, ADC, T1c)***	40	27	67.5%		Oligodendroglioma	Astrocytoma	Glioblastoma
Oligodendroglioma	14	12	85.7%	Oligodendroglioma	12	0	2
Astrocytoma	10	4	40.0%	Astrocytoma	5	4	1
Glioblastoma	16	11	62.5%	Glioblastoma	5	0	11
***Not-cropped (FLAIR, ADC, T1c)***	40	24	60.0%				
Oligodendroglioma	14	11	78.6%	Oligodendroglioma	9	2	3
Astrocytoma	10	2	20.0%	Astrocytoma	4	3	3
Glioblastoma	16	11	68.8%	Glioblastoma	3	0	13
***Segment addition (T1c, T1c, se)***	40	24	60.0%				
Oligodendroglioma	14	11	78.6%	Oligodendroglioma	11	1	2
Astrocytoma	10	2	20.0%	Astrocytoma	6	2	2
Glioblastoma	16	11	68.8%	Glioblastoma	5	0	11
Image only (T1c, T1c, Zero *)	40	20	50.0%				
Oligodendroglioma	14	4	28.6%	Oligodendroglioma	4	6	4
Astrocytoma	10	2	20.0%	Astrocytoma	3	2	5
Glioblastoma	16	14	87.5%	Glioblastoma	0	2	14
***Slice pooling (ADC(n* − 1*), n, (n* + 1*))***	40	17	42.5%				
Oligodendroglioma	14	6	42.9%	Oligodendroglioma	6	3	5
Astrocytoma	10	4	40.0%	Astrocytoma	3	4	3
Glioblastoma	16	7	43.8%	Glioblastoma	8	1	7
***Individual slice treatment (ADC, ADC, ADC)***	40	22	55.0%				
Oligodendroglioma	14	8	57.1%	Oligodendroglioma	8	2	4
Astrocytoma	10	1	10.0%	Astrocytoma	6	1	3
Glioblastoma	16	13	81.3%	Glioblastoma	3	0	13

Note: ResNet34 and second strategy of transfer learning were used to generate all result. Bold parts represent titles of different combinations. Zero *: zeroed matrix of same shape.

**Table 5:** Summary of classification results for different strategies of transfer learning on test sets.

Combinations of 3 Image Modalities	n (Total)	n (Correct)	Accuracy	Subtype	Confusion Matrices		
***ResNet34 with Transfer method A*** ***(ADC, FLAIR, T1c)***	40	27	67.5%		Oligodendroglioma	Astrocytoma	Glioblastoma
Oligodendroglioma	14	12	85.7%	Oligodendroglioma	12	0	2
Astrocytoma	10	4	40.0%	Astrocytoma	5	4	1
Glioblastoma	16	11	68.8%	Glioblastoma	5	0	11
***ResNet34 with Transfer method B*** ***(ADC, FLAIR, T1c)***	40	24	60.0%				
Oligodendroglioma	14	10	71.4%	Oligodendroglioma	10	1	3
Astrocytoma	10	2	20.0%	Astrocytoma	7	2	1
Glioblastoma	16	12	75.0%	Glioblastoma	4	0	12
***ConvNext tiny with Transfer method B*** ***(ADC, FLAIR, T1c)***	40	22	55.0%				
Oligodendroglioma	14	8	57.1%	Oligodendroglioma	8	2	4
Astrocytoma	10	2	20.0%	Astrocytoma	6	2	2
Glioblastoma	16	12	75.0%	Glioblastoma	4	0	12
***VIT-base with Transfer method B*** ***(ADC, FLAIR, T1c)***	40	17	42.5%				
Oligodendroglioma	14	12	85.7%	Oligodendroglioma	12	2	0
Astrocytoma	10	2	20.0%	Astrocytoma	8	2	0
Glioblastoma	16	0	0.0%	Glioblastoma	0	0	0
***ResNet34 not pretrained*** ***(All images)***	40	20	50.0%				
Oligodendroglioma	14	8	57.1%	Oligodendroglioma	8	0	6
Astrocytoma	10	3	30.0%	Astrocytoma	4	3	2
Glioblastoma	16	9	56.3%	Glioblastoma	6	3	9

## Data Availability

The data in this article is not provided for protecting the privacy of the patients. If necessary, the author can be contacted via email at xiongdh0801@163.com.

## Supplementary Materials

The following supporting information can be downloaded at: https://www.mdpi.com/article/10.3390/diagnostics12123063/s1, Figure S1: Structure of the network.; Figure S2: Train and loss curve of final selected model; Table S1: Imaging parameters of MRI acquisition protocols.
