# Supplementary material for: Noninvasive Classification of Glioma Subtypes Using Multiparametric MRI to Improve Deep Learning
Source: Diagnostics (Basel). 2022 Dec 6;12(12):3063. doi: 10.3390/diagnostics12123063 (PMC9776470; doi:10.3390/diagnostics12123063)
Supplement: Supplementary file 1 [file diagnostics-12-03063-s001.zip › diagnostics-2027942-supplementary.pdf]

Table S1. Imaging parameters of MRI acquisition protocols (Ingenia and Ingenia CX 3T, Philips Healthcare).

Acquisition order	Sequence	TR(ms)	TE(ms)	Flip angle	Acquisition matrix	Thickness(mm)	Acquisition time
1	sagittal 3D-T1W	5.9	2.7	8°	240×240	1	2 min 8 s
2	axial T2W	4000	122	90°	384×384	6	2 min 8 s
3	axial FLAIR	8000	120	—	352×140	6	2 min
4	axial DWI	2668	88	90°	152×122	6	32 s
5	sagittal post-contrast 3D-T1W	5.9	2.7	8°	240×240	1	2 min 8 s

Note: 3D=three-dimensional; FLAIR=fluid-attenuated inversion-recovery; DWI=diffusion weighted imaging; pCASL=pseudo-continuous arterial spin labeling; APT=amide proton transfer.

Implementation Details

Our implementation was based on the pytorch. During training, the probability of each patient sample belonging to the wild-type or mutant IDH class was computed with networks. The weights of the network were optimized via a stochastic gradient descent algorithm. The objective function used was weighted cross-entropy. The weights were set according to the number of samples in each classification. We used a training batch of size 16, chose the Adam optimizer as the optimizer, the learning rate was set to 1e-4, and the weight decay was set to 1e-8. We also used learning rate decay, which is also valid on the adam optimizer\cite[2017Decoupled], here exponential continuous decay function with the decay rate set to 0.98.

The convergence process of different models is different, so in order to better compare the performance of different models, we use the early stopping method. The precedure is stopped when the validation loss does not decrease for 20 consecutive epochs. Since the model is unstable in the early stage of training, we start using early stopping after 5 epochs.

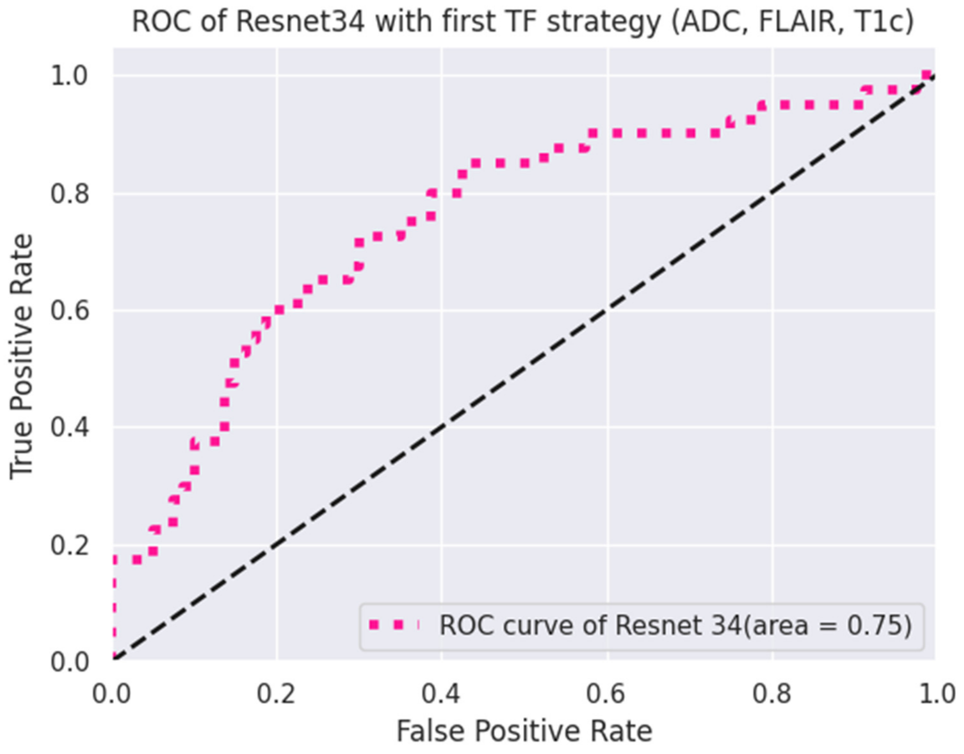
ROC of Resnet34 with first TF strategy (ADC, FLAIR, T1c)
True Positive Rate
False Positive Rate
ROC curve of Resnet 34(area = 0.75)
0.0
0.2
0.4
0.6
0.8
1.0

A.

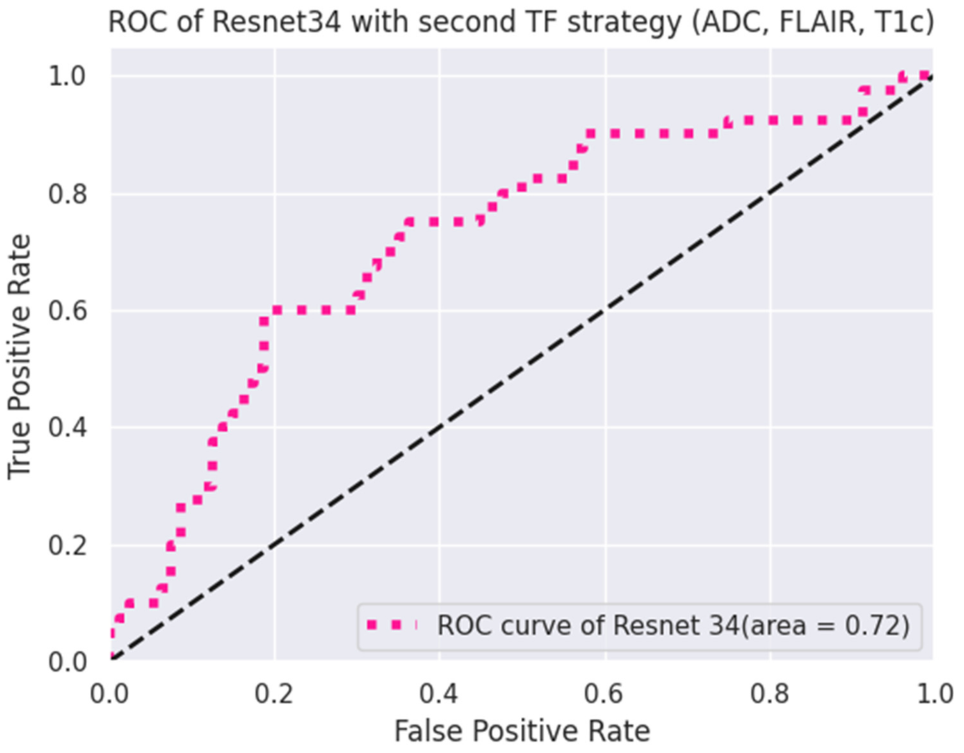
ROC of Resnet34 with second TF strategy (ADC, FLAIR, T1c)
True Positive Rate
False Positive Rate
ROC curve of Resnet 34(area = 0.72)
0.0
0.2
0.4
0.6
0.8
1.0

B.

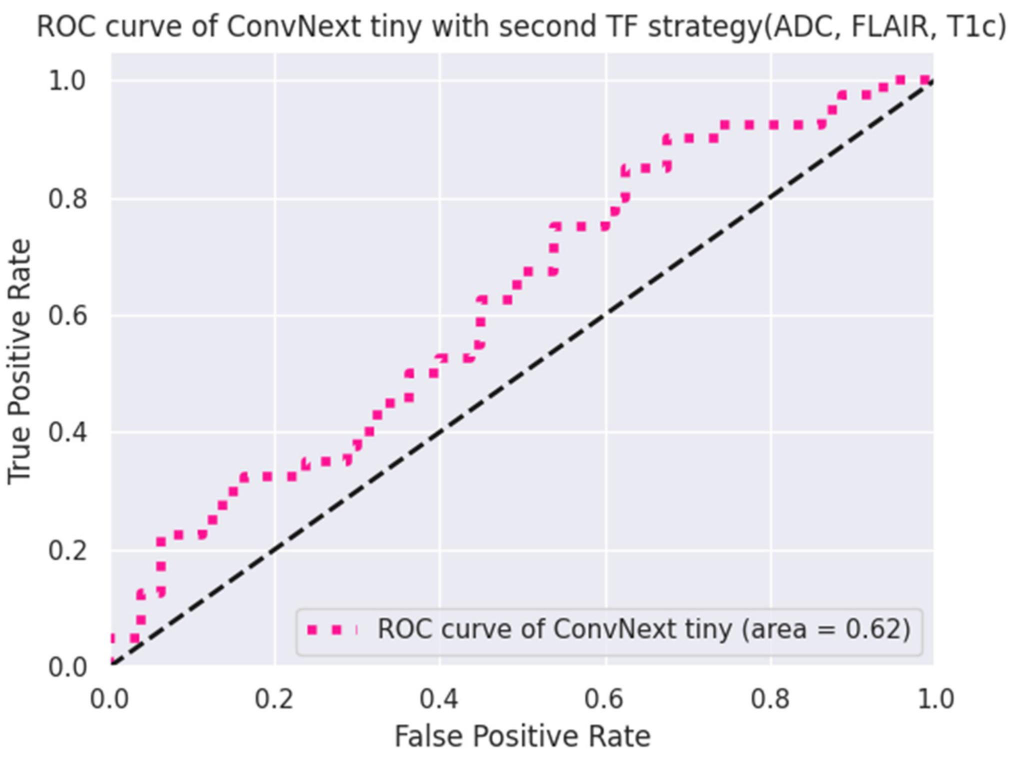

C.

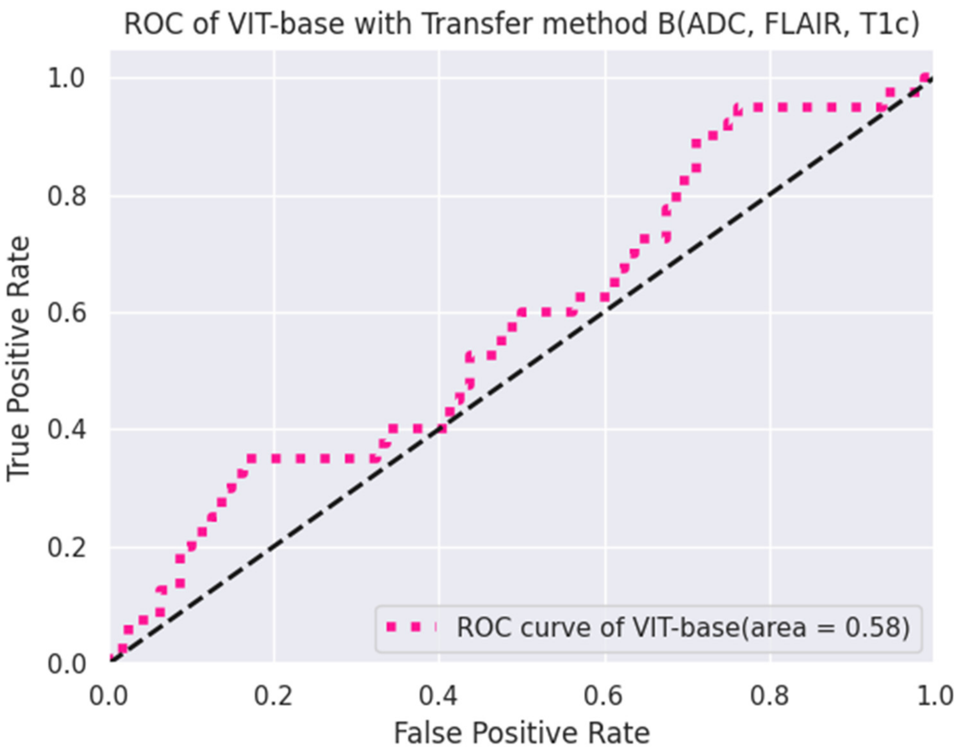

D.

Supplement Figure S1. ROC of different networks. (A)ROC of ResNet34 with first strategy of Transfer learning (TL). (B)ROC of ResNet34 with second strategy of TL. (C) ROC of ConvNext tiny with second strategy of TL. (D) ROC of VIT-base with second strategy of TL.

A.

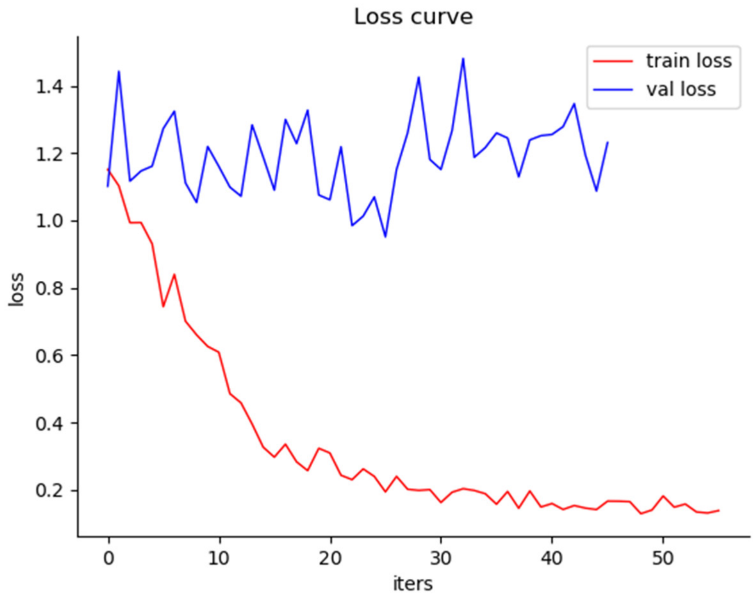

B.

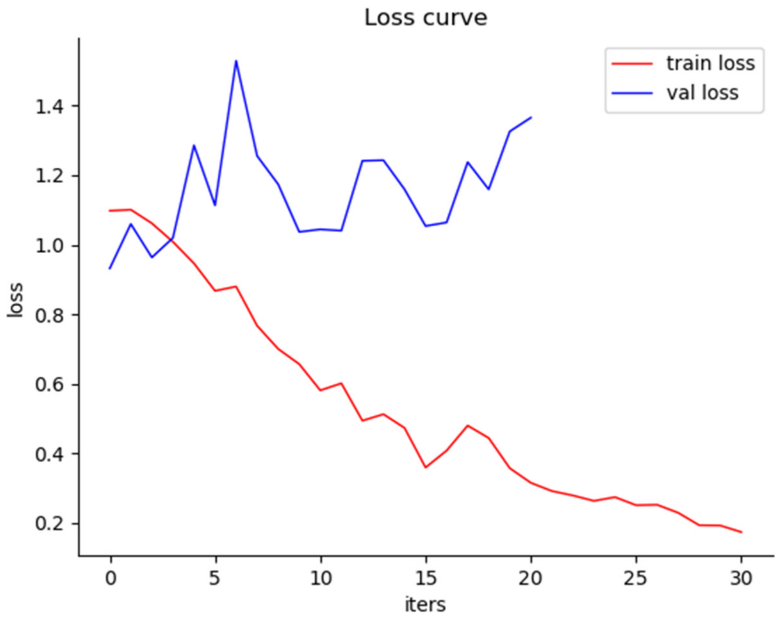

Supplement Figure S2. Train and loss curve of final model. (A) curve of ResNet 34, using both image and numeric data. (B) curve of ConvNext tiny, using both image and numeric data.
